# Current Artificial Intelligence (AI) Techniques, Challenges, and Approaches in Controlling and Fighting COVID-19: A Review

**DOI:** 10.3390/ijerph19105901

**Published:** 2022-05-12

**Authors:** Umar Albalawi, Mohammed Mustafa

**Affiliations:** 1 Faculty of Computing and Information Technology, University of Tabuk, KSA, Tabuk 71491, Saudi Arabia; mmustafa@ut.edu.sa; 2 Industrial Innovation and Robotics Center, University of Tabuk, KSA, Tabuk 71491, Saudi Arabia

**Keywords:** COVID-19, artificial intelligence, machine learning, medical imaging, dataset, open resource, genome and protein sequences, robotics, drones, global health

## Abstract

SARS-CoV-2 (COVID-19) has been one of the worst global health crises in the 21st century. The currently available rollout vaccines are not 100% effective for COVID-19 due to the evolving nature of the virus. There is a real need for a concerted effort to fight the virus, and research from diverse fields must contribute. Artificial intelligence-based approaches have proven to be significantly effective in every branch of our daily lives, including healthcare and medical domains. During the early days of this pandemic, artificial intelligence (AI) was utilized in the fight against this virus outbreak and it has played a major role in containing the spread of the virus. It provided innovative opportunities to speed up the development of disease interventions. Several methods, models, AI-based devices, robotics, and technologies have been proposed and utilized for diverse tasks such as surveillance, spread prediction, peak time prediction, classification, hospitalization, healthcare management, heath system capacity, etc. This paper attempts to provide a quick, concise, and precise survey of the state-of-the-art AI-based techniques, technologies, and datasets used in fighting COVID-19. Several domains, including forecasting, surveillance, dynamic times series forecasting, spread prediction, genomics, compute vision, peak time prediction, the classification of medical imaging—including CT and X-ray and how they can be processed—and biological data (genome and protein sequences) have been investigated. An overview of the open-access computational resources and platforms is given and their useful tools are pointed out. The paper presents the potential research areas in AI and will thus encourage researchers to contribute to fighting against the virus and aid global health by slowing down the spread of the virus. This will be a significant contribution to help minimize the high death rate across the globe.

## 1. Introduction

Since the first appearance of SARS-CoV-2 (COVID-19), researchers in different fields have raced against time to contain this global pandemic. Machine learning (ML) is one of the research fields where researchers have been actively involved in determining different potential obstacles [1] and providing solutions to fight against the spread of the virus.

COVID-19 is an infectious disease caused by a severe (acute) respiratory syndrome called coronavirus. The name comes from the visual appearance of the solar corona as seen when using an electron microscope, which looks like a crown. Due to the global spread of this virus, it has become very important to explore, understand, and model novel treatment and diagnostic approaches to nullify this threat for future generations.

Corona viruses (CoV) are a large family of viruses that lead to illnesses that range from the common cold to severe diseases like severe acute respiratory syndrome and the Middle East respiratory syndrome [2]. The novel CoV is a newer strain of CoV that had not previously been identified. The incubation period of COVID-19 ranges from 1 to 14 days [3]. 

In this review, an in-depth analysis is performed with an eye to fill the gaps in current review papers and to keep researchers up-to-date by providing newly developed techniques. Accordingly, the paper highlights several technical studies that have been performed on artificial intelligence (AI) research articles and the solutions that have been proposed. 

## 2. Global Status

**Figure 1:**
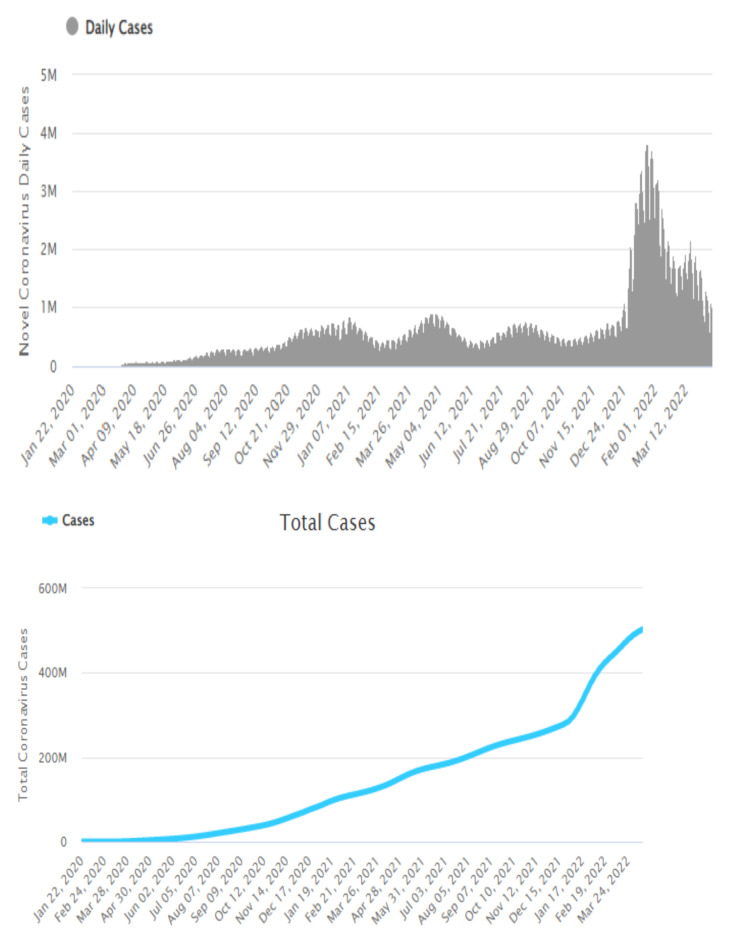
Global statistical report for COVID-19 (to Mar 2022)—world meters.

COVID-19 led to a massive worldwide outbreak, leading patients to suffer respiratory illness and acute respiratory distress syndrome. By April 2022, the disease has resulted in 6,219,002 deaths and 503,349,008 infected individuals to date, representing a rapid spread of this infectious disease. Countries like China, Italy, the United States of America, Germany, France, and Spain have reported substantial numbers of deaths due to COVID-19. As shown in Figure 1, it is a fast-spreading disease and as a result, was declared a Public Health Emergency of International Concern by the WHO in 2021 [1]. The wide spread of COVID-19 globally poses a significant threat to human health internationally. It is diagnosed via a real-time reverse transcriptase-polymerase chain reaction, which has caused limited supplies of tests and increased laboratory environment requirements. This has led to the delayed diagnosis of infected individuals, which makes it more difficult to prevent the spread of this infection, most specifically in regions experiencing an epidemic [4]. Clinical diagnosis factors, clinical symptoms/signs, laboratory findings, and travel history aid in the fast prediction and straightforward diagnosis of COVID-19. This helps to isolate and control the epidemic promptly in regions experiencing severe epidemics. Computed tomography (C.T.) is a key component of disease prediction in affected persons.

The more specific, accurate, and faster prediction of COVID-19 in the preliminary stages plays a crucial role in shaping self-quarantine and treatment guidance, which have high significance for patient prognosis and the control of the disease, and provides public health security. In Wuhan, individuals have to take a chest C.T. to identify the changes in the body and severity/cause of severe pulmonary pneumonia. This puts a considerable burden on the medical staff. This disease suddenly causes shortness of breath, lung infection, and worse health conditions. High-resolution C.T. scans can help identify small regions of ground-glass opacity. In the initial stages, COVID-19 tests are performed using a chest C.T. with peripheral sub-pleural opacity. This consumes massive amounts of time when predicting the consolidation patterns of COVID-19 [5]. As things stand, missed cases can lead to a massive spread of the disease and poses a significant challenge with tremendous work and risk being involved in achieving diagnostic accuracy. This increases the potential risk of missed diagnoses due to lesions being small.

The WHO has established a timeline of various organizations’ response activities to generate necessary information. It will update the status of the timeline on a regular basis. Significantly, the country-based information is provided to the WHO by its state members. As reported by the WHO, trials involving hydroxychloroquine (HCQ) as a COVID-19 treatment were stopped. The principal investigators said that the decision was based on proof from solidarity trials [6]. A Cochrane review was completed on the hydroxychloroquine trials. The data were released with an announcement that HCQ does not affect outcomes in reducing the hospital mortality of COVID-19 patients [7,8]. No random observation was performed; instead, HCQ was started in the initial phase of treatment with the physician’s supervision. This decision was made based on the solidarity trial’s outcomes, and it cannot apply to the use of HCQ in pre-or post-exposure to patients with COVID-19. Based on a report published in June, the WHO welcomes clinical results from the U.K. that demonstrate that dexamethasone, a corticosteroid, can save the lives of patients who are critically ill with COVID-19 [9,10]. For patients on ventilators and patients who need oxygen, treatment can reduce mortality by one-third. Therefore, mortality can be cut by one-fifth based on the preliminary findings of the WHO [11]. The WHO follows local authorities regarding clusters of COVID-19 cases, and officials from national health commissions share the initial investigators’ details. The WHO has offered technical assistance and support regarding COVID-19 clusters and investigations are underway [12,13]. With the report released on 3 June 2020, the executive group recommends continuing the solidarity trail, which includes HCQ [14]. The monitoring and data safety committee continue to closely observe the safety of all patients tested with solidarity trials. Faster prediction leads to lower mortality. As suggested by the WHO, the clinical data is under investigation and various researchers have attempted to provide solutions to the COVID-19 pandemic [15].

Since its appearance, due to the speed of the disease outbreak, researchers in different fields have been in a race against time to stop this serious worldwide threat. AI is not an exception and researchers in the field have paid special attention to different potential obstacles [16]. They have looked into how to provide solutions in the hope of fighting the disease. In fact, AI has garnered the attention of various researchers and diverse experimentation has been carried out and various approaches adapted for predicting, tracking, and monitoring the spread of the virus [17].

AI and machine-learning solutions could have a significant impact on fighting the disease. For instance, machine learning techniques have been used intensively in the study of different diseases in terms of protein analysis, forecasting, and prediction, and paving the way towards vaccines and antivirals. An example of such a disease is the seasonal flu.

From this perspective, a huge number of AI approaches (including disease forecasting, surveillance, expected peak, and spread models) have been proposed and developed for several diseases—including the seasonal flu [18,19,20,21,22], which is relatively similar in its symptoms to COVID-19. The most notable teams that also contributed to this area are the Massachusetts Amherst (UMass Amherst) and Carnegie Mellon researchers. For example, researchers from these two universities have used AI in the early detection and surveillance of influenza activity as this could have a significant role in our preparedness for the disease. Such forecasting will help in tracking the disease before, during, and after disease season. AI has been also used for developing different vaccines for different strains of influenza virus by firstly examining virus mutation. With the use of AI, it becomes possible to personalize vaccines according to age or population. Models were also developed to predict peak time, expected peak date in hospitals, and detection through cough sounds.

However, their significant achievements do not mean that they are ready to be utilized for COVID-19 because each disease has its unique features, which may not be shared with others. Accordingly, the current surveillance systems for tracking the seasonal flu, for example, might not suit COVID-19 because the spread rate and characteristics of the diseases are different. This is especially true for COVID-19 as little is known about the virus, its potential method of infection, and how it behaves in the human body.

Compared to peer diseases such as Severe Acute Respiratory Syndrome (SARS-CoV), EBlOa Virus (EBOV), and the Middle East Respiratory Syndrome CoronaVirus (MERS-CoV), COVID-19 does not show a high rate of fatalities such as EBOV, but it is more transmissible than the seasonal flu. This shows that these diseases have very different characteristics. For instance, infected individuals can transmit the disease even before symptoms begin to appear, making it challenging to contain outbreaks. This confirms our conclusion that AI-based techniques that are developed for specific diseases may not be valid for other diseases.

In contrast, the absence of AI-based techniques when studying diseases may slow our progress towards finding an adequate vaccine and understanding how the virus mutates. Consider the case of HIV (Human Immunodeficiency Virus), which causes AIDS (Acquired ImmunoDeficiency Syndrome). When HIV was first discovered, AI-based techniques were extremely limited. In fact, early AI-based systems were mainly based on massive programming models and on decision rules that were often prepared by experts in specific domains. In addition, computing power during those days was an issue, unlike today, due to the absence of cloud computing. As a result, prevention measures, for example, were still primitive and thus disease detection, prediction, and surveillance could not be performed unless trained persons in the medical field conducted investigations. Another problem was the virus mutation. Since there were no AI techniques such as those we have today, only experts could discover virus mutations. With the use of bioinformatics, genomic sequencing data, and machine learning, it is now possible to identify types of mutations and how the virus evolves with a high degree of accuracy.

Currently, AI is used to carry out many tasks related to HIV. For example, Ref. [23] makes use of machine learning (XGBoost algorithm in particular) to identify people who are living with HIV and those with a high likelihood of contracting the disease. The reported results show that XGBoost has significantly improved HIV positivity identification. Examples also include the studies presented in [24,25,26].

For these reasons, artificial intelligence can play a significant role in fighting diseases and can achieve high performance [27] (i.e., disease prediction using medical imaging). Chest C.T. data, for example, can be used to predict COVID-19 infection faster with reduced computational complexities [28]. Therefore, modeling artificial intelligence-based methods for COVID-19 detection is beneficial. To formulate a proper solution to this pandemic and provide a productive model of the disease, AI modeling approaches should be adopted.

A tremendous number of studies and reviews in different domains have been conducted so as to reduce mortality rates. For example, Ref. [29] gives a good overview of papers related to COVID-19. It surveys many recent studies related to efforts to fight the disease. Review topics are mainly focused on silico methodologies and tools for virus structure prediction, vaccine design, virtual drug screening, phylogenetic analyses, and natural source-derived chemicals. AI and machine learning approaches were included in the study to some extent but from a biologist’s point of view. The issue of what types of available datasets can be used to perform specific AI tasks related to COVID-19 was briefly covered in the paper. We believe that one way to help researchers is to alert them to the datasets, platforms, and tools that are available for research, along with their natures. 

Another valuable review is provided in [30]. It reviews several studies that make use of different AI and machine learning techniques. However, the study was focused on the epidemiology forecasting of COVID-19 using AI techniques. This is different from what is presented in this paper as our review here covers many different topics—including the available datasets, image processing, medical imaging, the use of sensors and robots, etc.

## 3. COVID-19 in Computer Vision

**Figure 2:**
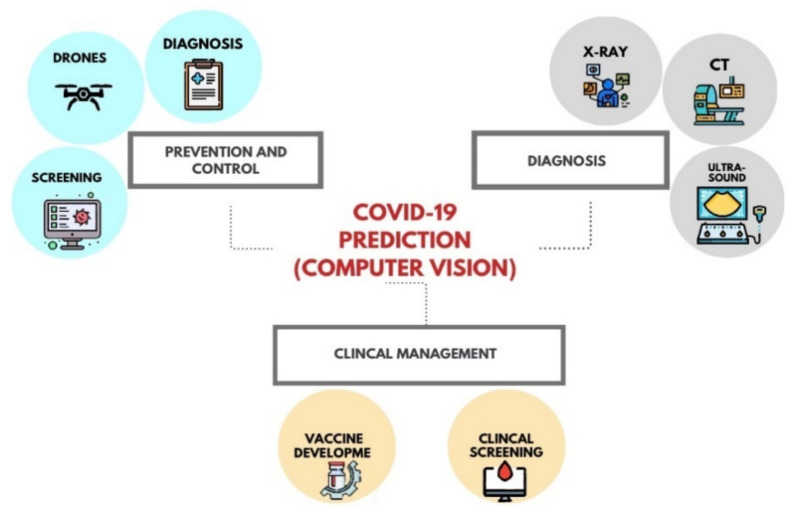
Classification of computer vision approaches to COVID-19.

Computer vision is one of the main areas of AI that has been investigated in the context of COVID-19. Reference [31] is a valuable study that surveys many articles related specifically to COVID computer vision-based techniques. In that survey, COVID vision-based methods are classified into three main categories, these are diagnosis, disease control and prevention, and clinical management. The first class, diagnosis, is also classified into three sub-classes: X-ray, CT, and ultrasound. The second class, prevention, is categorized into three sub-classes: diagnosis, drones, and screening. Finally, the clinical management category is divided into patient screening and vaccine development. Figure 2 depicts the computer vision approach to the classification of COVID-19.

## 4. Diagnosis through CT and X-ray

The major problem in COVID diagnosis is to identify whether a potential patient is infected or not using lung images (CT scans or X-ray images, for example) [32,33]. 

The study in [34] proposed to automatically segment CT images in infected patients using the UNet++ segmentation model, which utilizes deep learning techniques. In CT scans, significant features often include lesion distribution, consolidation, nodules, etc. [35,36]. Hence, valid areas in images can easily be detected through segmentation. The reported experiments showed that the model, which was trained with more than 40,000 images, had an accuracy of more than 95%.

Different from this work, Ref. [37] preprocesses lung input images to segment them, and then the problem was handled as a multi-class classification problem that could be managed through COVNet to automatically extract visual features. In particular, one interesting feature of this study is that it attempted to classify whether pneumonia was caused by COVID-19 or other diseases like the flu. With the use of a relatively small number of CT scan images, the results indicated that the sensitivity was 90%. 

The work in [38] uses a similar technique in which the classification task involved three classes instead of two. In that work, a deep learning model based on DRE-Net (Deep Relation Extraction) was employed. The results were promising.

COVID diagnosis has also been tested using X-ray images. In contrast to CT scans, X-ray imagery technology is readily available and does not require highly trained experts. Motivated by this fact, the study in [39] developed a new architecture for deep learning called COVID-Net with the network trained using X-ray images. The model utilizes the pre-trained REsNet-50 to provide fine-tuned diagnosis results. In fact, the developed architecture and the model are one of the most valuable contributions to COVID computer vision. The model accuracy was greater than 92% when it was trained with more than 16,000 chest X-ray images. A similar approach with a different architecture was also developed by [40,41] and their accuracies were found to be relatively high. In particular, the former study combined seven different deep learning models that were based on DCNN architectures.

Recently, several review papers on the use of AI models in general and the use of CT-scan and X-ray images in particular in fighting the COVID-19 have been published [42,43]. The tasks are varied and significant progress has been achieved. Several insights can be gleaned from the results. First, the use of medical images in detecting and diagnosing COVID-19 is the most studied task. However, it should be noted that the majority of studies make use of CT scans and X-ray images and that the use of MRI and ultrasound is relatively rare [44,45]. The classification tasks were mainly binary (infected with COVID or not) [46,47], with some exceptions that attempted to add a pneumonia category [48].

The employed classifiers were mainly Convolutional Neural Networks (CNN). This is intuitive because of the ability of CNNs to provide good results [49,50]. A considerable number of studies employed the use of pre-trained models like MobileNet, VGG, and ResNet. Additional pre-trained models such as InceptionResNetV2 and Xception [51] were also used. Some studies attempt to apply transfer-learning models to pre-trained models using CNNs [52,53].

The use of traditional machine learning models such as logistic regression, random forest, generative adversarial networks, and Support Vector Machines (SVM) is extremely rare in the literature. However, some studies have combined these approaches with the use of a CNN and a pre-trained model. In fact, the best-reported results for classification tasks on CT and X-ray images of COVID-19 were achieved by such combined approaches [54,55,56].

## 5. Prevention in Computer Vision

An example of the use of computer vision in disease prevention is the work of [57], which uses recognition models with deep learning to automatically recognize mask-wearing. The achieved accuracy was 95% when using a dataset of gathered images named Masked Face Detection Dataset (MFDD) [58].

Infrared thermography techniques like fever and forehead temperature screening have also been tested for COVID detection [59]. Some studies also incorporate this technique into mobile-based applications [60].

Computer vision-based techniques have also been used in prevention via screening. The screening process is considered an inexpensive process; however, it assists in predicting and managing the symptoms of COVID-19, specifically in large and populated regions. A robot named spot has been used to observe signs such as respiratory rate, skin temperature, and oxygen saturation in the blood that are required for conventional screening. Monochrome cameras have been used for tracking skin temperature, R.R., H.R., and so on. The authors of [7] used drones to sense patients’ temperature and predict physical signs like respiratory rate, heart rate, sneezing, and coughing. Similarly, machine learning and deep learning approaches have been adopted to perform screening to help physicians make appropriate decisions at critical times. Misty methods are used to measure capabilities and human–robot interaction. Thermal cameras provide accuracy within 0.5 °C. When compared to traditional diagnosis methods and screening, there are some benefits to the adoption of robots. They can work effectively in risky conditions, gradually reducing unnecessary contact between patients and staff. This improves the safety of healthcare workers. Various sensors such as microphones, speakers, I.R., cameras, and thermal sensors are involved in the screening process [61]. With the advancements in newer technologies, screening helps with the sharing of essential information and the construction of networks to deal with the pandemic.

U.V. technology represents one method of sterilization. Surfaces are directly exposed to U.V. light to disinfect them. Similarly, a general method of disinfecting spaces and areas with U.V. involves adopting robotic cards, handheld devices, and dirty plates in hospital rooms. During the pandemic, various kinds of disinfection robots were modeled to fight against coronavirus [61]. These models are mixed with commercial forms with mobile platforms and U.V. lamps. This lamp takes roughly 30 min to cover 380 square meters at a speed of about 0.1 m/s. Mobile platforms are provided with U.V. robots. Commercial robots are well-known products with complete sensing ability to handle the working environment.

In spite of the significant help that can be achieved via the use of the above mentioned technologies, there is a real limitation that prevents us from gaining the maximum benefits from them. This is especially true in third-world countries, whose health systems’ capacities and resources are not able to afford expensive medical devices. During the spread of COVID-19, we saw the limited capacity of the economic and medical health systems in developing and third-world countries, which could not afford to take care of the rising numbers of potential patients and often suffered from acute shortages in medical devices, physicians, and trained operators for new technologies. In fact, the capacity of the health systems in developing (and even in first-world) countries is still far from that required to deal with the high number of patients to be handled—COVID-19 patients—and the system cannot absorb all suspected cases. As a result, resources may be limited when considering advanced machines. Hence, the use of sophisticated technologies such as drones and screening technologies may not be applicable all the time, especially in low- and medium-income countries.

## 6. Clinical Management and Treatment in Computer Vision

The enormous scale of vaccination programs caused some unapproved treatments to be used. The symptoms of COVID-19 need to be treated efficiently based on the patient’s clinical condition [62]. The improvement of patients’ clinical condition is likely with automatic practices and the assistance of computer vision. For instance, the classification process is conducted based on the severity of the disease. The mapping of images helps predict the activation region, which helps monitor the disease progression and severity detection [62]. Infected regions must be visualized, and severity is identified with superior patient care and disease management.

## 7. Telemedicine and COVID-19

The use of telemedicine has also been investigated. Telemedicine involves the use of recent technology by experts to treat patients as an alternative to in-person appointments with physicians. Such a solution would fit the needs of developing and third-world countries, whose health systems’ capacities and resources are not able to afford a high number of cases. Hence, telemedicine could have a significant impact on world health in different countries where resources are limited. Usually, telemedicine is adapted with AI to conduct contact tracing by using heath applications on any smartphone or wearable device [63]. Globally, 58% of countries are using telemedicine to deal with COVID-19 [64].

One example of a telemedicine approach is the so-called Chatbot. Technically, a chatbot is the natural development of what is known as *Question Answering Systems* in Natural Language Processing (NLP). As the term indicates, in a question answering system there is a user need that is often submitted to an NLP system to provide the best response. However, in automatic conversational agents, the technology goes further to combine these techniques with artificial intelligence. On the one hand, NLP can be used to analyze conversational data and understand human needs (often questions). On the other hand, AI is utilized to identify hidden associations in different contexts and to formulate the best responses to different needs.

Recently, chatbots have been widely and intensively used. One of the most vital fields for the use of chatbots is the healthcare and medical domains. It is known that the medical sector assists billions of patients every year across the world. However, the sector is very time-sensitive and is closely linked to intensive human interactions (i.e., doctor–patient conversations). In addition, the sector has a lack of technicians and physicians. With an eye on these vital facts, many attempts have been made to utilize chatbots and conversational agents in healthcare systems. Accordingly, they have been used in scheduling doctor appointments, guiding emergency cases by locating nearby clinics, reducing the time by recoding observations and assessing cases before accessing hospitals, and explaining and providing necessary information about medication [65]. For example, during the outbreak of COVID-19, the World Health Organization (WHO) offered a chatbot via WhatApp to provide information about the disease. The same organization also launched a chatbot on Facebook Messenger in order to combat misinformation and provide accurate information about COVID-19 [66]. Other countries use the same chatbot service technology for the same purpose of making information about the pandemic available via telephone and/or instant messaging.

After the outbreak of COVID-19, Battineni and his team [67] proposed a sophisticated framework for an artificial intelligence chatbot to serve as an instant messaging consultant whenever specialists were not available, especially in rural areas. Accordingly, the agent helps to evaluate potential cases who have been exposed to coronavirus. Following this, the chatbot recommends the required action. Two major phases are identified to achieve this goal. First a request analysis phase and then a return response phase. The first phase is centered around evaluating disease severity through a predefined questionnaire. Depending on potential patient answers, an Artificial Intelligence Markup Language (AIML) logic is used to extract symptomatic keywords to assess the underlying disease. AIML is a language used for creating natural language agents to provide interactions. In the second phase, the chatbot produces generated text from its knowledge base [67]. Nevertheless, the chatbot is just a pilot framework and is still under development. Gadewar and his team followed the same steps in [53]. They utilized an AI agent that is based on if-else logic to train the chatbot for health consultancy in the case of insomnia and sleep disorders [68]. 

From the previously reviewed studies, it can be seen that there is no unified standard data with which we can say some techniques are better than others. This is especially true for studies from the early days of the pandemic. Additionally, after researchers in different domains began to develop new techniques, new and diverse datasets are becoming available every day. This is a real challenge that may affect the AI community because unless these datasets become standardized we cannot tell which technique is the best. For this reason, in this paper, we provide some of the standard datasets that have been developed to fight COVID, each of which is primed towards a specific target. This can help AI researchers to develop their methods using a single, unified dataset.

## 8. Detection of COVID-19 through Cough Sound Recognition

Detection of COVID-19 has also been attempted through the cough sound [55,63,69,70,71,72]. The underlying assumption here is that the lung sound of those who are infected has some unique features that can be easily used for the purpose of identifying whether the sound is a symptom of COVID-19 infection. The task is often challenging because of the noise that may occur in real environments (like a clinic). This is true if we consider the fact that the datasets are primarily collected using traditional stethoscopes. Thus, a sophisticated signal processing technique is required as it plays a vital role in the task. Some studies have also incorporated their developed systems of cough sound recognition for infected individuals into computer-aided programs and mobile applications [63]. In [63] specifically, the work involved the multi-classification task of differentiating between four classes (asthma, COVID-19, bronchitis, and innocent cough).

## 9. AI in COVID-19 Drug and Vaccine Development

AI approaches have also been used in vaccine and drug development. However, they are often employed in some underlying sub-tasks of vaccine development. In particular, the majority of studies involving AI and vaccine/drug development attempted to find chemical ingredients and molecules that could be used for drug discovery. The study in [73] used Reinforcement Learning (RL) and deep Q learning to produce lead compounds for coronavirus protease target 3CLpro inhibitors and to predict molecules and lead components. A similar approach was also followed by [74], in which a Recurrent Neural Network (RNN) and RL models were used. Other studies, such as [75], used traditional methods such as neural networks and Naïve Bayes to identify drugs by testing compounds and selecting the drugs that affect the spike protein as candidates. The ChemAI deep learning model, which is based on a Long Short Term Memory (LSTM) model, was also used by [76] to examine how 3CLpro protease can be resisted using molecules. The work in [77] examined the possibility of predicting vaccines using a machine learning model and so-called reverse vaccinology (RV) approaches and through an analysis of pathogen genome bioinformatics. Thus, the virus sequence and all its proteins were analyzed to predict biological features. In the model, a set of machine learning models was used to predict levels of proteins in coronavirus. It included the use of K-nearest neighbors (KNN), re-enforcement learning, support vector machine, and XGBoost.

## 10. Epidemiological Models vs. AI Models

Epidemiological spread models, such as the classic Susceptible Infectious Recovered (SIR) model, help us to quantify the complex epidemic dynamics and the total number of confirmed cases associated with a specific disease (e.g., COVID-19). The models also help us to study the parameters (as functions) that allow individuals to infect one another (i.e., transition between susceptible to infected compartments) by estimating, for example, the probability of this transition using Bayes’ theorem [78,79,80]. For instance, recent epidemiological models allow us to quantify infected individuals’ profiles and the severity of the disease in them. These results suggest that the modeling of these probabilities can be carried out through machine learning and AI models. 

On the other hand, machine learning and artificial intelligence approaches can achieve good results, even with minimal steps for data preprocessing, including missed predictors and outliers. Additionally, they can easily capture nonlinearity in the relationship between responses and predictors. Nevertheless, machine learning depends on static datasets, while the nature of epidemiological data essentially evolves over time. Additionally, the major problematic issue in machine learning models is parameter optimization, which often requires experienced computer scientists to increase model performance. Moreover, it is not clear how the estimated data of the missed predictors affect performance, how they handle the specific features of outbreak data, such as in the case of COVID-19, or how they manage input data scales. This has led to recent research efforts to attempt to estimate parameters (e.g., Case Fatality Rate (CFR)) using machine learning models [78,79,80,81].

Inspired by these arguments, mathematicians, as usual, also contributed to the global fight against COVID-19. Several new mathematical models have been proposed. These models can represent new directions and paradigms that can be employed in AI techniques. Motivated by the fact that current epidemiological models often overestimate or underestimate the actual lethality rate, which is often used to describe the transition of patients from infected cases to deceased compartments, which in turn may affect nationwide testing and vaccination programs. Treibert and her team [82] developed a model to predict future the development of the pandemic in a specific area (i.e., a country). The developed model was based on SVICDR, which is a compartmental model, which in turn is derived from the susceptible infectious recovered (SIR) epidemiological model of Kermack and Mckendrick. The model is based on finding solutions to ordinary differential equation (ODE) systems. This is because the transitions of entities between several compartments can be mathematically expressed in terms of ODE systems. From this perspective, the model attempts to explore the significance of parameter (function) boundary modifications on predicted incidence rates while at the same time considering other parameters like vaccination and quarantine rates, trigonometric contact, etc. In the model, the transitions between the infected, vaccinated, intensive care, deceased, recovered, and susceptible compartments were considered. The results, which were tested using German pandemic data, showed that the model was able to identify asymptotic behaviors.

A similar approach was also followed in [83], but the model of the SIR was incorporated into a neural network to learn the parameters (i.e., quarantine function). The major task of the developed model was to build a diagnostic tool for quarantine policy evaluation after being quantified. The model was successful, and it has been implemented in over 70 countries as a global and independent diagnosis tool for quarantine modeling. The authors of [83] also developed a similar model to analyze quarantine’s role in managing the reproduction of coronavirus [78].

## 11. Computational and Open-Access Data Resources

With an eye on providing innovative opportunities to speed up the development of disease intervention and to provide tools for disease surveillance, several datasets and computational resources are being collected and compiled from different, diverse resources [84]. The major and common feature among all these datasets is their free availability to researchers and the fact that they have been made available to the research community. In other words, they represent open-access platforms and machine-readable resources for researchers in different domains, and most of them provide visualization tools that can give us some insights into the nature of the data. This important feature can be accredited to these platforms as they can be used to model the needed answers and to support the ongoing fight against the pandemic. Additionally, the provided datasets can be employed in different data mining and artificial intelligence tasks. For instance, there are many datasets that are collections of lung images of individuals who are infected with COVID-19. Using such datasets will help researchers to devise new algorithms for early detection of the disease and for checking its severity. Another example of available datasets is bioinformatics and genomic sequencing collections. Such datasets can be used to observe virus mutations by identifying which genomes change over time. A third example is the statistical epidemiological datasets, which provide us with up-to-date information about disease statistics. Such datasets can be employed for the purpose of predicting disease peaks in a specific country, for example. Epidemiological datasets can also be used by AI researchers for disease forecasting, spread prediction, peak time prediction, classification, expected hospitalizations, healthcare management, heath system capacity, etc. From this perspective, we believe that it is important in this study to provide researchers with some insights into the available datasets and platforms that can be used to develop novel AI-based techniques to fight epidemic diseases. In this review, we scanned the datasets that have been made available for researchers in different domains, including those in AI. Informally, we can divide these datasets into clinical trial data, epidemiological statistical data, digital image data, genomics data, and natural language processing-related data. The subsequent subsections discuss these types of datasets and computational resources.

## 12. Clinical Trial Data

By the term clinical trial data, we mean any study in which volunteers attempt to help tackle a specific research question related to a disease. Since COVID-19 spread quickly, several organizations raised many challenging questions that could only be answered through clinical trial data. Examples of challenging questions include what is the best combination of currently available drugs that can help fight COVID-19 infection? What is the adequate combination of chemical structures in potential vaccines that can provide us with a good candidate? What is the suitable dosage that can protect our bodies? Therefore, clinical trial data can provide researchers with valuable results and could have a significant impact on new discoveries and conclusions. For instance, the study provided by [85] attempts to investigate whether the loss of smell and taste senses is a symptom of COVID-19 infection or not. In [86], the researchers used lung CT scan images to discover the severity of lung damage in infected patients. The answer to the question of whether the TR-PCR test for COVID-19 will be affected by other diseases in potential patients is provided in [87]. Some researchers have attempted to study the impact of the test on the nasopharyngeal system. The same project also studied the effect of the disease on liver damage. Other researchers have investigated pregnant women who tested positive by PCR during labor or pregnancy and how this impacted the testing and the blood cord [88].

It is notable that innumerable clinical studies can be and/or are being conducted in countries across the world. The described studies are just a small sample and as the disease is still under analysis, a tremendous and very diverse number of clinical data are available. In order to make accessing these different studies and their datasets easier, the U.S. National Library of Medicine [89] provides a weekly updated database in CSV format for COVID-19-related clinical studies that were and/or are being conducted around the world with more than 380,700 included in mid-July 2021. Another example of such a valuable repository is AccessClinicalData@NIAID, which is provided by the American National Institute of Allergy and Infectious Diseases (NIADID) and is available at [90]. In terms of AI, such data can be coupled with powerful deep learning models and can be mined through data mining approaches to discover any interconnections between different phenomena and the disease. Hence, there is an endless list of models that can be applied to these clinical trials.

## 13. Epidemiological Statistical Data

Another type of available and open-access dataset is epidemiological transactional data. This type of data includes useful and up-to-date statistical information. It is often used by researchers to predict and investigate specific phenomena such as COVID-19 surveillance, expected availability of hospital beds, and confirmed deaths. An example of such a dataset is the AWS (Amazon Web Service) data lake for COVID-19 analysis [91]. It is a valuable repository supported by some visualization tools for curated datasets related to COVID-19. 

There are many datasets in the repository available in CSV that are related to vaccine distribution, disease surveillance, available hospital beds, confirmed cases, and daily, weekly, or monthly death and testing data in the U.S. and/or around the world (including the famous John Hopkins statistics). A dataset for the Pfizer vaccine distribution across the United States is given in [92]. Similar datasets are also available at [93] by the United States Centers for Disease Control and Prevention (CDC). The repository also illustrates important statistics and distributions for data such as healthcare data, serology test surveillance (test for antibodies in the blood), disease surveillance in general, emergency department visits, hospital capacity, etc. For Europe, the European CDC provides the geographic distribution of COVID-19 cases worldwide [94]. One advantage of the latter repository is that it is updated daily. There are also some repositories that specialize in aggregating epidemiological data (and genomic sequencing of the virus) during outbreaks of disease—including COVID-19, such as the Outbreak project [95]. It is evident that statistical epidemiological datasets can be utilized for the purpose of monitoring diseases’ forecasting, spread prediction, and peak time prediction using AI and data mining techniques. 

## 14. Digital Image Data

**Figure 3:**
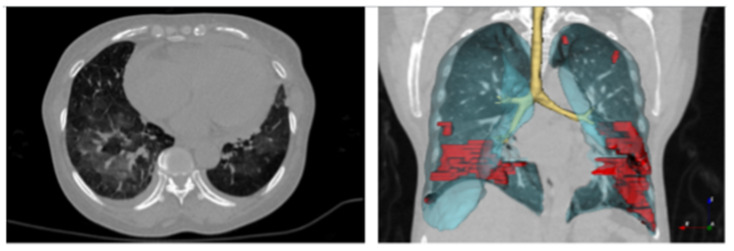
Samples of digital images. Source: [97].

Image datasets have also been made available to researchers in different fields. The COVID Digital Pathology Resource (COVID-DPR) is a set of histopathologic image samples of the disease [96]. It includes tissue images of the lung, liver, and kidney biopsy and autopsy samples, as well as heart images, before and/or after being infected with COVID-19. In the same repository, other images for similar diseases like SARS are also available for comparison. Figure 3 shows examples of such digital images taken from [97].

Another valuable repository for digital images is the COVID-19 datasets on The Cancer Imaging Archive (TCIA), which is provided by the Cancer Imaging Program (CIP), National Institute of Health (NIH) and is available at [98]. In fact, the program utilizes its Cancer Imaging Archive for COVID19 radiology and digitized histopathological images. The datasets are diverse and include radiology, CT, and X-ray images. An example of this type of dataset is available at [97], in which there are CT scans of 632 patients infected with COVID-19 in the initial stages and 121 serial and sequential CT scans of 29 patients. Digital imaging is a valuable resource for AI models. They play an increasingly vital role in the early detection of different diseases, including COVID-19. For this disease, in particular, the medical literature reports that chest imaging for those potentially infected patients often shows some specific lung findings. Accordingly, AI and digital image processing can be utilized for such purposes.

## 15. Genomic Data

This is one of the highly important types of datasets because it tackles the sequencing data of COVID-19 (SARS-CoV2) and the genomic sequence for all its strains, translated proteins, and nucleotide sequences. Such genomic repositories also include methods for visualization and analytic tools for generating multiple sequences of alignments of interest. Recently, the use of large-scale genomic data mining and AI approaches has led to a great explosion in genomic data. For instance, classification learning has been used to predict genomic models of different groups of biological cells, e.g., under immunologic constraints for different patients in diverse classes or for predicting sequence similarity against a well-identified task. Moreover, large-scale genomic data mining techniques and AI computational approaches can also be used for performing combinatorics of genetic code and genes in a DNA sequence and in cellular differentiation for capturing variants as well. From this perspective, AI and data mining researchers were given a good opportunity to examine their methods as this pandemic unfolded.

Examples of genomic platforms and repositories include the Broad Terra Cloud Commons for Pathogen Surveillance (available at [99]), the GenBank Nucleotide Sequences (available at [100]), and the COVID-19 Genome Sequence Dataset (available at [101]). The latter repository, for example, includes sequencing genomes related to coronavirus (SARS-CoV-2). The Global Initiative on Sharing Avian Influenza Data (GISAID), available at [102], also provides very rich genomic sequencing data, as well as vaccine developments, the genomes of virus variants, and diagnostic tests for COVID-19. Chemical structure and protein structure data have also been gathered and compiled. The Coronavirus3D collection [103] illustrates changes in the sequenced genomes of COVID-19 variants in terms of mutations, lineages, and their different combinations in different geographical areas.

Table 1 illustrates some variants of COVID-19 (SARS-CoV2), cited from [103], and the spike proteins of some coronavirus variants, cited from [104]. The collection of 3D Print Models of SARS-CoV-2 Virions and Proteins is another corpus that consists of a structural protein data bank in 3D format. Features such as spike receptors, main protease, and papain-like protease proteins are found in the collection. The dataset of CAS COVID-19 antiviral candidate compounds contains approximately 50,000 chemical substances from antiviral drugs and their associated compounds [105]. The dataset is populated in a suitable structure for data mining and machine learning algorithms.

## 16. Natural Language Processing-Related Data

Besides the described dataset samples, Kaggle and others [106] provide a valuable resource that contains more than 500,000 scholarly articles on COVID-19. The dataset is available and presents a challenge for the natural language processing and artificial intelligence research communities. Their stated objective is to produce new knowledge and insights. The COVID-19 Open Research Dataset and AI Challenge (CORD-19) [107] is another resource containing over 500,000 scholarly articles, including over 200,000 with full text, about COVID-19, SARS-CoV-2, and related coronaviruses. These resources can help the global research community to apply recent advances in natural language processing and other AI techniques to generate new insights to support the ongoing fight against this infectious disease.

## 17. Testing COVID-19

An enormous number of testing samples are frequently required to test for the virus, due to its contagiousness and wide diffusion among populations. Thus, the rapid increase in the testing capacity is essential for the health system [10]. A device known as a rapid automated volume enhancer is designed to save physicians time and reduce lab workers’ workload. Nearly 4000 samples are collected with this device for the preliminary process. Similarly, a cobot YuMi device has been developed to improve serological testing and automate the screening process in order to reduce human effort. With this device, roughly 450 samples can be collected. Some researchers have integrated a robotic liquid handler with a polymerase chain reaction for automating the testing process and increasing the total capacity, i.e., 100 samples per day [8]. Another device known as an ultra-high throughput mass spectrometer is used to perform pipetting and collect samples from 190 patients per day. The testing of COVID-19 is conducted in various forms depending on the country’s ability to invest a considerable amount in research. Various solutions have been suggested to test for COVID-19. However, some testing processes give better results than others, as is shown in Table 2. It is feasible to increase and improve the testing flexibility to create better protocol variations in the future.

## 18. Evaluation Process

The first line of defense against the virus is conducted with proper clinical management. It is carried out by predicting the severity of the patient’s condition with immediate medical actions, i.e., ventilator support. The disease progression score is provided to categorize the type of the patients’ infection. This is termed a corona score. This score is computed with the infection measurements and disease severity based on C.T. images. It evaluates the disease progression over a specific period and evaluates the volumetric summation of activation mapping. It helps radiological evidence as a tool to differentiate the necessary condition of the patients [108]. In some cases, deep learning approaches and cameras are used to study the respiratory patterns (abnormal conditions) for an extensive scale screening process with disease infection, which is more appropriate [108]. The respiratory simulation model is designed to manage the real-world data gap and an enormous amount of sample training. There are six different respiratory patterns, known as central apnea, Cheyne–Stokes, eupnea, tachypnea, boots, and bradypnea, that can be used to identify a patient’s clinical illness. The anticipated model is used to categorize the respiratory patterns with better prediction accuracy.

## 19. Research Challenges of Using Computing Approaches

COVID-19 has had a significant impact on the social and economic lives of a substantial number of people. The adoption of computing approaches helps to track COVID-19 with approaches that vary from image processing to bio-informatics. For effective execution, the collection of sample data is extremely essential, whether open-source or closed-source. The adoption of open-source data is more prominent for COVID-19 prediction. Thus, the integration of open-source data and intelligent computing plays a substantial role in diagnosis using some medical images like CT or chest X-ray scans [61]. The utilization of diverse computing approaches helps with COVID-19 diagnosis with the available images and helps to overcome the constraints of reverse transcriptase-polymerase chain reaction test kits; however, the transmission of the virus is evaluated via epidemiological analyses. Moreover, the extraction of social data offers a socio-economic analysis. The utilization of open-source data and intelligent computing models offers effectual outcomes for predicting the trends of the COVID-19 pandemic. However, there are still issues to confront when utilizing COVID-19 open-source data.

The preliminary factor is the challenge of the improvement of medical image accuracy for clinical practice. A patient can show a positive test result but a chest examination remains the primary identification source [62]. The medical images remain the secondary source of prediction. Other challenges are the provisioning of correlation between the RT-PCR and radiographic tests. The challenge related to contactless development is to protect physicians from tinfected individuals. Most of the prevailing datasets are smaller and this leads to lower prediction accuracy. The accuracy of deep learning approaches can be improved for larger dataset analysis. The analysis of the epidemiological outcomes is not so accurate. Thus, the prediction accuracy is determined as the fundamental factor for researchers attempting to predict the virus’ dissemination.

## 20. Impact of Data on COVID-19 Research 

The development of prediction models for epidemics is extremely crucial to provide perspectives on the spread of the virus, and the outcomes need to be measured. For prediction, clinical data are required rather than online data. The epidemiological approaches are more efficient and constant. Generally, social connections are not so certain. With large-scale analysis, some enhanced epidemiological approaches like DL and ML approaches have captured the attention of researchers for the construction of these models. 

Motivated by the fact that about one-third of COVID-19 patients are likely to transmit to critical cases, [109] developed a model to predict mortality risk in patients who are severely infected with COVID-19. Around 2800 medical records were compiled from patients in Tongji Hospital, Wuhan, China during January and February 2020. In the study, the researchers used the supervised XGBoost classifier due to its ability to identify patterns and the fact that it features choices. As the study is still in progress, the initial results reported that the model almost reaches 100% accuracy concerning death and is able to detect survival with 90% accuracy.

Some have proposed [110] the use of a Kalman filter with linear models to predict the spread of coronavirus. Using the dataset of [111], which shows total deaths, recoveries, and confirmed cases per location, Kremer showed that the model is able to predict the spread for the next 30 days with accurate results using one-day Kalman prediction, but the model is less able to predict infection peaks. Inspired by the fact that the total number of individuals infected is often much higher than the officially announced figures, Ref. [112] in their study to forecast COVID-19 disease utilized what they called a susceptible–infected–recovered–dead (SIRD) model simulation to predict three-week forecasts of the evolution of the disease outbreak. The results, which were tested using data collected from the 11th of January to the 18th of January 2020, showed that the estimated parameters are accurate to some extent for predicting disease spread and estimating actual numbers of infected, recovered, and death cases. Other studies [113] have focused on potential medications by analyzing proteins in order to aid vaccine and antiviral development. The majority of such studies, which need a relatively strong foundation in the medical field, make use of convolutional neural networks. Other researchers [34,114] have analyzed images of X-rays and CT scans to accurately diagnose COVID-19 patients. The aim here was to shorten the time spent on testing.

**Figure 4:**
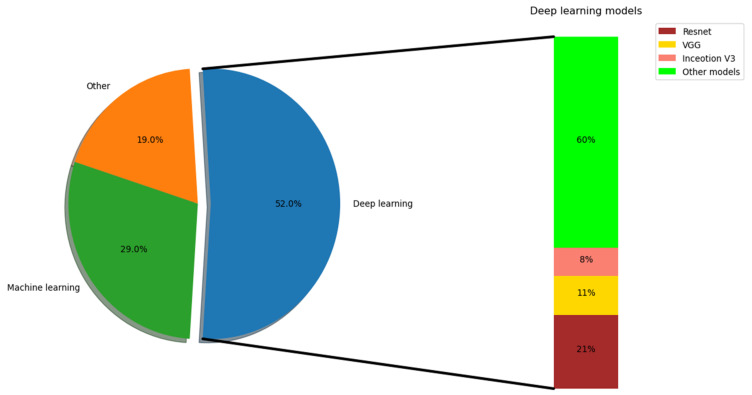
Contribution of various techniques to COVID-19 prediction.

A machine learning technique pretends to enhance approaches, giving superior generalization capability and prediction consistency. The adoption of ML during data processing provides explicit information. Time series data is often used as the input for ML algorithms as they show variations over time. AI shows its significance for improving the relationship between spatial attributes and attribute extraction in [17]. DL approaches are competent at predicting quantitative metrics and feature extraction using CT images and can perform various comparisons with quantitative measures. The investigators need to acquire categorization strategies with multi-class and hierarchical perceptions. There are diverse kinds of inputs, such as lung image datasets, datasets for detection purposes, and data from publicly accessible datasets, which are used by DL and ML approaches for COVID-19 prediction. Diverse methodologies are used to predict COVID-19. They include DL and ML models for predicting related issues. Some in-depth analyses have been carried out by various researchers with statistical measures. Some CNN models are used for predicting COVID-19 with certain contributions, as shown in Figure 4. Similarly, for predicting COVID-19, robots are also associated with major advantages. Generally, robots work efficiently in risky environments with reduced unnecessary contact between the patients and medical staff. This improves the safety of healthcare workers [63]. A robot can work for a long time and relieves some of the pressure on nurses and doctors. For instance, robotic serological testing is a major help to lab technicians. Robots can thus improve throughput and efficiency. They can leverage the use of sensors to carry out multiple tasks simultaneously. Some are modeled specifically to predict COVID-19 with well-equipped sensors like microphones, thermal sensors, IR, speakers, cameras, and so on. Various multi-modal robots are modeled for initiating diagnostic functions. The application of robots is, however, limited due to their difficult technological development. With these technologies, robots can perform various tasks in a standalone manner and assist in sharing information and constructing the network model to deal with these pandemics. Due to their powerful abilities, robots are considered the predominant fighters in the battle against COVID-19. However, there are diverse challenges related to the adoption of robots. The taxonomy level must be raised constantly. Some robot-based sampling processes can eliminate contact between the patients and the medical staff. Teleoperations are needed to complete the sampling procedure. Thus, in the case of a fully automated process, some components are merged with AI and computer vision. Similarly, reliability must be guaranteed with various applications [63].

Sensor technologies and their components are required. Robots are complex systems that help us attain better performance. Temperature screening is essential in hospitals and other places. Hardware development is modeled to examine vital physical signs from the provided sensor data. It is a non-trivial task to extract the essential information from the sensors and attain appropriate accuracy. Additionally, the construction of the complete network model is also a great task [17,115]. Most robots work as an individual unit, and some may provide suspicious reports. These robots need to be connected to the IoT, AI, 5G, or cloud technology. Some networks are formed by leveraging the accessible information to examine the pandemic situation in the community or in buildings such as hospitals. These networks may offer effective guidance for policymakers. The adoption of effectual models for predicting COVID-19 trends is accelerated and receives medical attention during the reduction of major exposure. The exposure to surgical robots is reduced when patients recover more quickly. The retrieval of essential care with quicker recovery enhances the treatment results. Tele-procedures and robots for healthcare help the world to grow towards the next level of treatment processes. However, there are diverse challenges that must be met. Within a limited time period, massive economic strides and techniques are needed to provide telehealth for COVID-19.

## 21. What Has Been Learned So Far?

In spite of the significant advancements through the use of artificial intelligence models in several aspects of fighting COVID, there is a long way to go in order to gain the maximum benefits. One of the major obstacles to current research is the massively noisy data, which has been collected through different techniques [42]. It should also be noted that a huge amount of fake news has been collected when building prediction models or extracting useful knowledge. Such behavior could result in invalid and inadequate training and hence could produce significantly biased results that could even bias the announced goal of the research. The problem becomes even more severe when the results are used in the government sector, for example, in CDC centers. There is another problem related to data that completely contradicts the aforementioned arguments. This is the problem of the availability of annotated, insufficient, and standard large-scale data. It is true that massive datasets are available, but we do not know much about the disease and thus the data changes quickly and can quickly become out-of-date. We also noticed that there are a lack of time-series datasets, which are often used in epidemiological models.

One more limitation related to medical imaging is the lack of negative samples (infected patients). It is true that such medical imaging techniques are not accessible all the time for many clinics and medical centers in many developing countries. As a result, negative examples in current image sets are considerably small compared to positive ones. This skew in the data may also bias machine learning models.

Another major limitation, which has also been indicated by [42,43], is the gap between the AI researchers, who are largely computer scientists, and medical researchers. The use of AI in medical fields usually requires a deep understanding of medicine, for example, the use of AI in drug and vaccine development, virology, mutation cycles, genomic data, bioinformatics, etc. Unless these communities work together, we cannot gain the maximum benefits of AI approaches to different tasks involved in fighting the disease.

## 22. Discussion

In their efforts to fight the COVID-19 pandemic, which threatens human lives in all countries around the world, researchers in different disciplines have been working together with the hope of stopping this health crisis.

One of the main communities that has contributed to this campaign is AI researchers, who quickly began to apply recent techniques in machine learning and AI in order to make up for the inherent limitations of human knowledge related to the disease. As a result, AI-powered techniques have been quickly applied and/or developed for a broad range of tasks at different stages of disease progression. Examples include disease detection, prediction, surveillance, the use of drones and robotics, remote communication, telemedicine, etc. One way to contribute to the fight against this disease is to provide researchers and experts with a quick review of what techniques and what datasets and tools are available to them. This will allow the research community to better understand, track, and fight diseases using AI techniques. We have seen that AI-powered techniques have proven themselves when fighting the disease and significant breakthroughs have been made, but there are still many limitations.

This paper reviews the state-of-the-art AI-based techniques used to fight COVID-19. It is evident that the virus has drastically accelerated the importance of AI and that the latter can play a significant role in controlling the outbreak. Its promising impact has drawn the attention of researchers. A considerable number of the developed approaches have already been applied in our daily lives and we immediately saw their real impact. Various control and prevention mechanisms have been adopted to facilitate the solutions and have proven their success. For instance, several approaches and paradigms are proposed to trace epidemiological statistical details, such as the prediction of epidemiological peaks, the dynamic simulation of time-series forecasting, number of cases, vaccine distribution, disease surveillance, available hospital beds, confirmed cases, and daily, weekly, or monthly death and testing data. Various AI, deep learning, and machine learning approaches have also been adopted to analyze and recognize CT and X-ray images, especially of the lungs, and these techniques have proven their effectiveness in the early detection of the disease. Nevertheless, we should also note that a significant number of studies, especially those that are based on clinical trial data, are still indicative rather than conclusive. This is because their results are still changing and hence require analysis in order to provide solid solutions and results that will contribute to reducing the relatively high numbers of deaths and minimizing infection rates, and saving human lives. *Examining* vaccine alternatives to discover possible treatments and genomic sequencing have also been exploited through AI techniques. Such techniques can have significant outcomes for researchers in medical fields.

In this paper, the available computational and data-access resources are summarized. Tremendous effort has been put into building open access and shared computational resources and the samples have been gathered from various sources very quickly. It is known that AI approaches are data-driven and that they are mainly influenced by the quality of the adopted dataset. The natural language processing (NLP) community has also been influenced by the significant amount of data generated. Various tools, methods, and techniques have been developed to analyze generated text in several languages. Such NLP techniques can help us to analyze, summarize, cluster, and classify substantial data and texts regarding the disease. They can also help us to defeat rumors and opinion polarity in specific contexts.

AI-based methodologies have proven themselves in the fight against COVID-19 and significant achievements have been earned, but there are many limitations to their use. For example, wearing-mask image detection, Bluetooth technology, social distancing, patient movement tracking, and discovering potential infectious patients are still inaccurate and far from achieving their stated goals. However, these limitations may be inherent to Bluetooth technology itself, which may fail to detect nearby cellphones. The progress of the AI in medical imaging has already been proven in the case of many diseases, but for COVID-19 it seems that more investigation and techniques are needed. In our opinion, this is mainly due to the fact that the science still needs to be studied and better understood. Once it is completely understood, many digital image processing, segmentation, and classification techniques can be utilized.

We cannot say that the use of AI in clinical trials still has limitations as such studies are always performed by pharmaceutical companies and their results are often not revealed. However, it is clear that researchers in AI need to focus on such areas, including vaccines, drugs, and dosage analysis, as well as DNA and genomic sequence data analysis. In fact, the research outcomes in this area are constrained by the lack of clinical study results in many cases.

The natural language processing NLP community also needs to come up with more convincing approaches for exploiting related articles in different languages. Thus, instead of utilizing the same techniques, new algorithms should be devised and developed. Audio cough sounds and signal processing can be also examined in detail since the majority of current studies in this area are inaccurate.

Robotics has also been intensively used in the battle against COVID-19. Since taking a sample via swabbing potentially infected individuals may lead to disease spread, robotics has been adopted for this crucial task to eliminate direct contact. Spot robotics have also been used to observe respiratory rate, temperature, etc. Others have been developed to reduce costs and the risk of disease infection. Drones have also been developed for monitoring crowds and mask images. Nevertheless, researchers in AI still need to provide new applications for robotics that minimize the risks to human lives. Eventually, we can say that this survey is considered a baseline for adopting AI approaches in future research.

## 23. Limitations

However, we believe that there are some limitations to this study. First, since there are a tremendous number of research papers in the field, it is impossible to cover the entire scope of these studies. This is especially true if we consider the fact that there is a global campaign underway to fight the disease and a great deal of data has been shared as part of this effort. As a result, many studies were not covered, but some have been reviewed. Second, the paper did not cover the alternative solutions provided for fighting COVID-19 in developing countries with limited economic resources and medical health systems. This means that many of the advanced techniques and approaches are impractical in such countries and rural areas. Therefore, most of the reviewed studies may only be applicable in specific regions and countries. Third, this work is also limited by the absence of any type of experimental evaluation of the reviewed techniques.

## 24. Conclusions

COVID-19 is one of the worst pandemics that humans have faced during this century due to its incremental acceleration of the number of both death and infection, which are relatively high. The disease also has a very negative impact on the global economy of the world. For these reasons, researchers in different fields attempt to fight the disease through their available tools. AI researchers are one of the most influential entities that contribute to the global campaign of fighting the disease using several approaches in different possible areas like screening, disease prediction, drug developments, diagnosis, etc.

This paper reviews the state-of-the-art AI-based techniques used to fight COVID-19. The paper provides a detailed summary of some representative research studies in different fields in which AI techniques are being used. Through this paper, a broad range of tasks have been described. 

From the review, it can be concluded that AI-based techniques and deep learning have proven to be helpful for fighting COVID-19 through different methods. Examples include but are not limited to, disease detection, prediction, surveillance, the use of drones and robotics, remote communication, telemedicine, robot usage, peak-time prediction, etc. AI methods also showed their vital role in assisting medical experts in vaccine discovery stages. Hence, the paper represents a quick review of what techniques and what datasets and tools are available to researchers.

## Figures and Tables

**Table 1:** Sample variants of COVID-19 (SARS-CoV2), cited from [103]. The dates of the variants are provided in the titles row.

Variant	Total	2021-05-11	2021-05-18	2021-05-25	2021-06-01	2021-06-08	2021-06-15	2021-06-22	2021-06-29	Recent Growth
B.1.617.2	4533	0.23	0.49	0.36	0.64	1.18	2.17	2.16	3.45	2.16
AY.2	133	0	0	0	0.42	0.76	5.28	1.35	5.24	1.35
B.1.621	145	0.33	0.16	0.8	0.48	1.23	3.79	1.57	0.61	0.61
B.1.315	94	0.31	0	0.05	0	2.57	1.49	0.89	1.42	0.89
C.36.3	204	0.67	0.63	0.47	0.86	0.62	1.19	0.82	0.65	0.65
B.1.1.318	656	0.6	0.2	0.37	0.8	0.72	0.82	0.91	0.7	0.7
B.1.448	297	0.22	0.2	0.19	0.33	0.69	0.77	0.81	0.55	0.55
B.1.1.523	51	0.35	0.8	0.57	0.55	0.75	0.75	0.94	0.29	0.29
P.1	17,101	0.66	0.81	0.54	0.75	0.91	1.19	0.69	0.77	0.69

**Table 2:** Treatment processes and implications.

S. NO	Treatment Method	Implications
1	With biophysical assays, spike protein of acute respiratory syndrome to host receptor	Cells virus related to host cells with spike glycoprotein, pre-fusion conformation, medical countermeasure development for prediction of COVID-19
2	Measuring corona score for disease progression screening and monitoring	Development of C.T. image and corona score is utilized to screen the illness of patients. For example, the score is measured at admission time and after disease recovery
3	Deep Learning and depth camera is utilized to categorize respiratory patterns.	Analyzing abnormal respiratory patterns can deal with large-scale screening for COVID-19 infection
4	Quantitative structural activities-based relational analysis with the assistance of deep learning	Utilization of potential rug discovery

## Data Availability

The study did not report any data.
